# 3-Benzyl-8-meth­oxy-2-sulfanyl­idene-1,2,3,4-tetra­hydro­quinazolin-4-one

**DOI:** 10.1107/S1600536812021794

**Published:** 2012-05-19

**Authors:** Rashad Al-Salahi, Mohamed Al-Omar, Mohamed Marzouk, Seik Weng Ng

**Affiliations:** a Department of Pharmaceutical Chemistry, College of Pharmacy, King Saud University, Riyadh 11451, Saudi Arabia; b Department of Chemistry, University of Malaya, 50603 Kuala Lumpur, Malaysia; c Chemistry Department, Faculty of Science, King Abdulaziz University, PO Box 80203 Jeddah, Saudi Arabia

## Abstract

The tetra­hydro­quinazole fused-ring system of the title compound, C_16_H_14_N_2_O_2_S, is roughly planar (r.m.s. deviation = 0.039 Å); the phenyl ring of the benzyl substituent is aligned at 78.1 (1)° with respect to the mean plane of the fused-ring system. In the crystal, two mol­ecules are linked by a pair of N—H⋯S hydrogen bonds about a center of inversion, generating a dimer.

## Related literature

For the synthesis, see: Al-Omar *et al.* (2004 ▶).
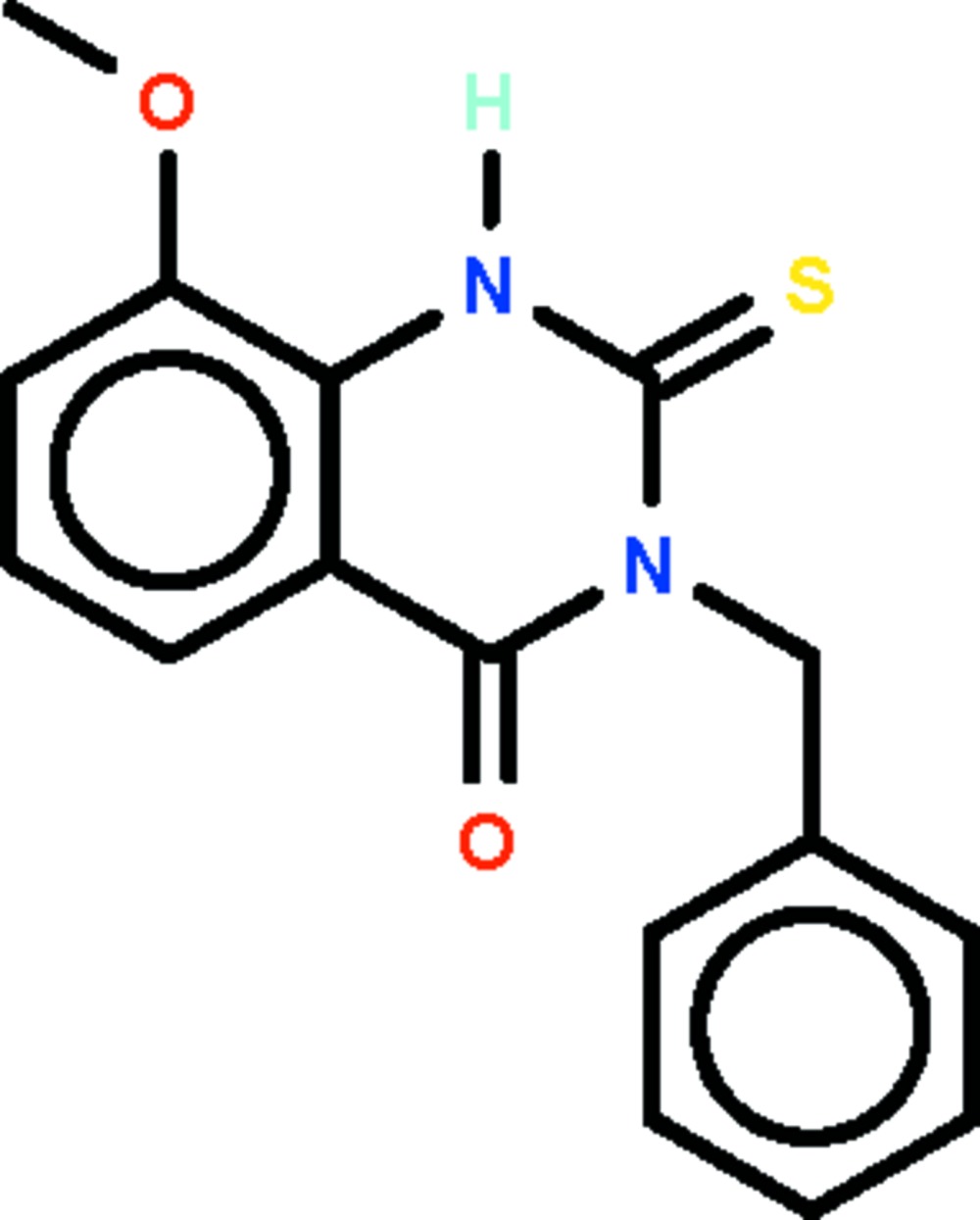


## Experimental

### 

#### Crystal data

C_16_H_14_N_2_O_2_S
*M* *_r_* = 298.35
Triclinic, 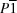

*a* = 6.3025 (5) Å
*b* = 10.8353 (5) Å
*c* = 11.0144 (7) Å
α = 101.728 (5)°
β = 102.419 (6)°
γ = 101.693 (5)°
*V* = 694.95 (8) Å^3^
*Z* = 2
Cu *K*α radiation
μ = 2.12 mm^−1^
*T* = 294 K
0.40 × 0.30 × 0.20 mm

#### Data collection

Agilent SuperNova Dual diffractometer with an Atlas detector
Absorption correction: multi-scan (*CrysAlis PRO*; Agilent, 2012) ▶ *T*_min_ = 0.484, *T*_max_ = 0.676
11149 measured reflections
2888 independent reflections
2714 reflections with *I* > 2σ(*I*)
*R*_int_ = 0.026

#### Refinement

*R*[*F* ^2^ > 2σ(*F* ^2^)] = 0.038
*wR*(*F* ^2^) = 0.114
*S* = 1.06
2888 reflections
195 parameters
1 restraint
H atoms treated by a mixture of independent and constrained refinement
Δρ_max_ = 0.22 e Å^−3^
Δρ_min_ = −0.40 e Å^−3^

### 

Data collection: *CrysAlis PRO* (Agilent, 2012 ▶); cell refinement: *CrysAlis PRO*; data reduction: *CrysAlis PRO*; program(s) used to solve structure: *SHELXS97* (Sheldrick, 2008 ▶); program(s) used to refine structure: *SHELXL97* (Sheldrick, 2008 ▶); molecular graphics: *X-SEED* (Barbour, 2001 ▶); software used to prepare material for publication: *publCIF* (Westrip, 2010 ▶).

## Supplementary Material

Crystal structure: contains datablock(s) global, I. DOI: 10.1107/S1600536812021794/bt5918sup1.cif

Structure factors: contains datablock(s) I. DOI: 10.1107/S1600536812021794/bt5918Isup2.hkl

Supplementary material file. DOI: 10.1107/S1600536812021794/bt5918Isup3.cml

Additional supplementary materials:  crystallographic information; 3D view; checkCIF report

## Figures and Tables

**Table 1:** Hydrogen-bond geometry (Å, °)

*D*—H⋯*A*	*D*—H	H⋯*A*	*D*⋯*A*	*D*—H⋯*A*
N1—H1⋯S1^i^	0.87 (1)	2.63 (1)	3.493 (1)	174 (2)

Symmetry code: (i) 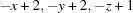.

**Fig. 1.**
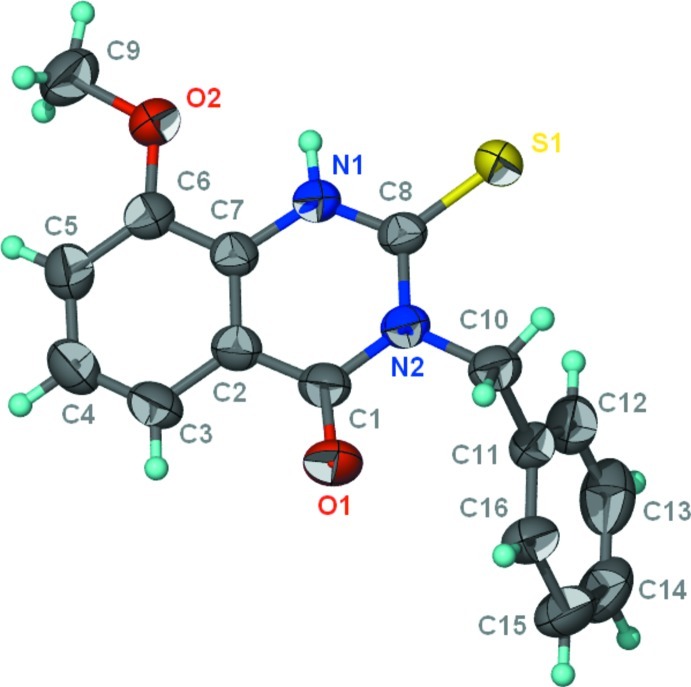
Anisotropic displacement ellipsoid plot (Barbour, 2001) of C16H14N2O2S at the 70% probability level; hydrogen atoms are drawn as spheres of arbitrary radius.

## supplementary crystallographic information

### Comment

The compound (Scheme I) was previously synthesized for a study of its antimicrobial activity. The background to this class of compounds is discussed (Al-Omar *et al.*, 2004). The tetrahydroquinazole fused-ring of C_16_H_14_N_2_O_2_S is flat; the phenyl ring of the benzyl substituent is aligned at 78.1 (1) ° with respect to the fused-ring (Fig. 1). Two molecules are linked by an N–H···S hydrogen bond about a center of inversion to generate a dimer (Table 1).

### Experimental

Benzyl isothiocyanate (10 mmol, 1.35 g), 2-amino-3-methoxybenzoic acid (10 mmol,1.67 g) and triethylamine (5 mmol, 0.51 g) in ethanol (30 ml) was heated under reflux for two hours. After cooling, the mixture was poured into ice-cold water. The resulting solid was filtered, washed with water and dried. Recrystallization from ethanol gave colorless crystals.

### Refinement

All H-atoms were located in a difference Fourier map. Carbon-bound H-atoms were placed in calculated positions [C–H 0.93 to 0.97 Å, *U*_iso_(H) 1.2 to 1.5*U*_eq_(C)] and were included in the refinement in the riding model approximation.

The amino H-atom was refined isotropically with a distance restraint of N–H 0.88±0.01 Å.

### Crystal data

**Table:**

C_16_H_14_N_2_O_2_S	*Z* = 2
*M**_r_* = 298.35	*F*(000) = 312
Triclinic, *P*1	*D*_x_ = 1.426 Mg m^−^^3^
Hall symbol: -P 1	Cu *K*α radiation, λ = 1.54184 Å
*a* = 6.3025 (5) Å	Cell parameters from 6739 reflections
*b* = 10.8353 (5) Å	θ = 4.3–76.5°
*c* = 11.0144 (7) Å	µ = 2.12 mm^−^^1^
α = 101.728 (5)°	*T* = 294 K
β = 102.419 (6)°	Prism, colorless
γ = 101.693 (5)°	0.40 × 0.30 × 0.20 mm
*V* = 694.95 (8) Å^3^	

### Data collection

**Table:**

Agilent SuperNova Dual diffractometer with an Atlas detector	2888 independent reflections
Radiation source: SuperNova (Cu) X-ray Source	2714 reflections with *I* > 2σ(*I*)
Mirror monochromator	*R*_int_ = 0.026
Detector resolution: 10.4041 pixels mm^-1^	θ_max_ = 76.7°, θ_min_ = 4.3°
ω scan	*h* = −7→7
Absorption correction: multi-scan (*CrysAlis PRO*; Agilent, 2012)	*k* = −13→13
*T*_min_ = 0.484, *T*_max_ = 0.676	*l* = −13→13
11149 measured reflections	

### Refinement

**Table:**

Refinement on *F*^2^	Primary atom site location: structure-invariant direct methods
Least-squares matrix: full	Secondary atom site location: difference Fourier map
*R*[*F*^2^ > 2σ(*F*^2^)] = 0.038	Hydrogen site location: inferred from neighbouring sites
*wR*(*F*^2^) = 0.114	H atoms treated by a mixture of independent and constrained refinement
*S* = 1.06	*w* = 1/[σ^2^(*F*_o_^2^) + (0.0717*P*)^2^ + 0.1365*P*] where *P* = (*F*_o_^2^ + 2*F*_c_^2^)/3
2888 reflections	(Δ/σ)_max_ = 0.001
195 parameters	Δρ_max_ = 0.22 e Å^−^^3^
1 restraint	Δρ_min_ = −0.40 e Å^−^^3^

### Fractional atomic coordinates and isotropic or equivalent isotropic displacement parameters (Å^2^)

**Table:**

	*x*	*y*	*z*	*U*_iso_*/*U*_eq_	
S1	0.84360 (6)	1.04442 (3)	0.64336 (3)	0.04455 (15)	
O1	0.15151 (18)	0.69504 (12)	0.56998 (12)	0.0543 (3)	
O2	0.8326 (2)	0.67738 (11)	0.27822 (11)	0.0509 (3)	
N1	0.7112 (2)	0.81847 (12)	0.46983 (11)	0.0376 (3)	
H1	0.829 (2)	0.8501 (18)	0.4456 (18)	0.051 (5)*	
N2	0.47878 (18)	0.84651 (11)	0.60456 (11)	0.0355 (3)	
C1	0.3257 (2)	0.72495 (14)	0.53999 (14)	0.0400 (3)	
C2	0.3924 (2)	0.64174 (14)	0.44134 (13)	0.0388 (3)	
C3	0.2672 (3)	0.51250 (15)	0.38286 (15)	0.0471 (3)	
H3	0.1396	0.4778	0.4067	0.057*	
C4	0.3356 (3)	0.43832 (15)	0.29020 (17)	0.0518 (4)	
H4	0.2549	0.3520	0.2525	0.062*	
C5	0.5233 (3)	0.48904 (16)	0.25080 (16)	0.0485 (4)	
H5	0.5643	0.4374	0.1860	0.058*	
C6	0.6480 (3)	0.61582 (14)	0.30800 (13)	0.0408 (3)	
C7	0.5842 (2)	0.69243 (13)	0.40631 (13)	0.0363 (3)	
C8	0.6691 (2)	0.89590 (13)	0.56926 (12)	0.0345 (3)	
C9	0.8974 (3)	0.60835 (19)	0.17369 (17)	0.0571 (4)	
H9A	1.0333	0.6606	0.1652	0.086*	
H9B	0.7802	0.5900	0.0957	0.086*	
H9C	0.9225	0.5279	0.1897	0.086*	
C10	0.4113 (2)	0.92622 (14)	0.70865 (14)	0.0397 (3)	
H10A	0.2568	0.9289	0.6768	0.048*	
H10B	0.5053	1.0148	0.7331	0.048*	
C11	0.4320 (2)	0.87257 (13)	0.82580 (13)	0.0401 (3)	
C12	0.6395 (3)	0.88228 (16)	0.90532 (16)	0.0501 (4)	
H12	0.7699	0.9204	0.8860	0.060*	
C13	0.6537 (4)	0.8349 (2)	1.01459 (18)	0.0662 (5)	
H13	0.7937	0.8417	1.0682	0.079*	
C14	0.4613 (5)	0.77823 (19)	1.04350 (18)	0.0698 (6)	
H14	0.4714	0.7466	1.1164	0.084*	
C15	0.2558 (4)	0.76845 (19)	0.96517 (19)	0.0666 (5)	
H15	0.1258	0.7299	0.9846	0.080*	
C16	0.2402 (3)	0.81568 (17)	0.85676 (16)	0.0524 (4)	
H16	0.0994	0.8091	0.8041	0.063*	

### Atomic displacement parameters (Å^2^)

**Table:**

	*U*^11^	*U*^22^	*U*^33^	*U*^12^	*U*^13^	*U*^23^
S1	0.0432 (2)	0.0386 (2)	0.0452 (2)	−0.00269 (15)	0.01814 (16)	0.00262 (15)
O1	0.0405 (6)	0.0597 (7)	0.0568 (7)	−0.0045 (5)	0.0229 (5)	0.0089 (5)
O2	0.0567 (7)	0.0465 (6)	0.0483 (6)	0.0031 (5)	0.0278 (5)	0.0052 (5)
N1	0.0370 (6)	0.0377 (6)	0.0364 (6)	0.0019 (5)	0.0151 (5)	0.0081 (5)
N2	0.0347 (6)	0.0383 (6)	0.0344 (6)	0.0057 (4)	0.0133 (4)	0.0109 (4)
C1	0.0357 (7)	0.0444 (8)	0.0375 (7)	0.0020 (6)	0.0101 (5)	0.0133 (6)
C2	0.0385 (7)	0.0392 (7)	0.0358 (7)	0.0029 (5)	0.0093 (5)	0.0112 (5)
C3	0.0429 (8)	0.0429 (8)	0.0493 (8)	−0.0027 (6)	0.0117 (6)	0.0124 (6)
C4	0.0542 (9)	0.0365 (7)	0.0540 (9)	−0.0016 (6)	0.0102 (7)	0.0059 (6)
C5	0.0572 (9)	0.0399 (7)	0.0443 (8)	0.0088 (6)	0.0140 (7)	0.0046 (6)
C6	0.0451 (7)	0.0410 (7)	0.0359 (7)	0.0069 (6)	0.0130 (6)	0.0109 (6)
C7	0.0381 (7)	0.0356 (6)	0.0329 (6)	0.0042 (5)	0.0086 (5)	0.0103 (5)
C8	0.0341 (6)	0.0378 (6)	0.0322 (6)	0.0060 (5)	0.0102 (5)	0.0125 (5)
C9	0.0654 (10)	0.0620 (10)	0.0522 (9)	0.0205 (8)	0.0308 (8)	0.0125 (8)
C10	0.0408 (7)	0.0401 (7)	0.0436 (7)	0.0107 (6)	0.0197 (6)	0.0131 (6)
C11	0.0488 (8)	0.0365 (7)	0.0373 (7)	0.0100 (6)	0.0194 (6)	0.0073 (5)
C12	0.0569 (9)	0.0475 (8)	0.0447 (8)	0.0141 (7)	0.0152 (7)	0.0070 (6)
C13	0.0895 (14)	0.0593 (10)	0.0468 (9)	0.0304 (10)	0.0055 (9)	0.0092 (8)
C14	0.1235 (19)	0.0547 (10)	0.0453 (9)	0.0316 (11)	0.0372 (11)	0.0198 (8)
C15	0.0951 (15)	0.0585 (10)	0.0608 (11)	0.0161 (10)	0.0477 (11)	0.0228 (9)
C16	0.0576 (9)	0.0531 (9)	0.0520 (9)	0.0094 (7)	0.0291 (7)	0.0149 (7)

### Geometric parameters (Å, º)

**Table:**

S1—C8	1.6818 (14)	C6—C7	1.407 (2)
O1—C1	1.2141 (18)	C9—H9A	0.9600
O2—C6	1.3582 (18)	C9—H9B	0.9600
O2—C9	1.4241 (19)	C9—H9C	0.9600
N1—C8	1.3475 (18)	C10—C11	1.5110 (19)
N1—C7	1.3867 (18)	C10—H10A	0.9700
N1—H1	0.868 (9)	C10—H10B	0.9700
N2—C8	1.3787 (17)	C11—C12	1.379 (2)
N2—C1	1.4074 (18)	C11—C16	1.385 (2)
N2—C10	1.4822 (17)	C12—C13	1.394 (3)
C1—C2	1.457 (2)	C12—H12	0.9300
C2—C7	1.3888 (19)	C13—C14	1.376 (3)
C2—C3	1.402 (2)	C13—H13	0.9300
C3—C4	1.367 (2)	C14—C15	1.364 (3)
C3—H3	0.9300	C14—H14	0.9300
C4—C5	1.394 (2)	C15—C16	1.384 (2)
C4—H4	0.9300	C15—H15	0.9300
C5—C6	1.378 (2)	C16—H16	0.9300
C5—H5	0.9300		
			
C6—O2—C9	117.68 (13)	O2—C9—H9A	109.5
C8—N1—C7	124.75 (12)	O2—C9—H9B	109.5
C8—N1—H1	116.9 (14)	H9A—C9—H9B	109.5
C7—N1—H1	118.4 (14)	O2—C9—H9C	109.5
C8—N2—C1	123.99 (12)	H9A—C9—H9C	109.5
C8—N2—C10	120.75 (11)	H9B—C9—H9C	109.5
C1—N2—C10	115.03 (11)	N2—C10—C11	112.13 (11)
O1—C1—N2	119.33 (14)	N2—C10—H10A	109.2
O1—C1—C2	124.39 (13)	C11—C10—H10A	109.2
N2—C1—C2	116.28 (12)	N2—C10—H10B	109.2
C7—C2—C3	120.22 (14)	C11—C10—H10B	109.2
C7—C2—C1	118.79 (12)	H10A—C10—H10B	107.9
C3—C2—C1	120.99 (13)	C12—C11—C16	118.91 (14)
C4—C3—C2	118.92 (14)	C12—C11—C10	121.20 (13)
C4—C3—H3	120.5	C16—C11—C10	119.87 (14)
C2—C3—H3	120.5	C11—C12—C13	120.01 (18)
C3—C4—C5	121.60 (14)	C11—C12—H12	120.0
C3—C4—H4	119.2	C13—C12—H12	120.0
C5—C4—H4	119.2	C14—C13—C12	120.3 (2)
C6—C5—C4	119.91 (15)	C14—C13—H13	119.9
C6—C5—H5	120.0	C12—C13—H13	119.9
C4—C5—H5	120.0	C15—C14—C13	119.94 (17)
O2—C6—C5	125.90 (14)	C15—C14—H14	120.0
O2—C6—C7	114.79 (12)	C13—C14—H14	120.0
C5—C6—C7	119.31 (14)	C14—C15—C16	120.18 (19)
N1—C7—C2	119.13 (13)	C14—C15—H15	119.9
N1—C7—C6	120.88 (12)	C16—C15—H15	119.9
C2—C7—C6	119.99 (13)	C15—C16—C11	120.70 (18)
N1—C8—N2	116.47 (12)	C15—C16—H16	119.6
N1—C8—S1	120.25 (10)	C11—C16—H16	119.6
N2—C8—S1	123.28 (10)		
			
C8—N2—C1—O1	−172.81 (13)	O2—C6—C7—N1	−3.0 (2)
C10—N2—C1—O1	1.76 (19)	C5—C6—C7—N1	177.19 (13)
C8—N2—C1—C2	8.29 (19)	O2—C6—C7—C2	177.30 (12)
C10—N2—C1—C2	−177.14 (11)	C5—C6—C7—C2	−2.5 (2)
O1—C1—C2—C7	173.21 (14)	C7—N1—C8—N2	−3.3 (2)
N2—C1—C2—C7	−7.96 (19)	C7—N1—C8—S1	176.82 (10)
O1—C1—C2—C3	−7.2 (2)	C1—N2—C8—N1	−2.82 (19)
N2—C1—C2—C3	171.67 (12)	C10—N2—C8—N1	−177.09 (11)
C7—C2—C3—C4	−0.8 (2)	C1—N2—C8—S1	177.03 (10)
C1—C2—C3—C4	179.61 (14)	C10—N2—C8—S1	2.76 (18)
C2—C3—C4—C5	−1.4 (3)	C8—N2—C10—C11	−112.55 (14)
C3—C4—C5—C6	1.7 (3)	C1—N2—C10—C11	72.68 (15)
C9—O2—C6—C5	3.9 (2)	N2—C10—C11—C12	72.02 (17)
C9—O2—C6—C7	−175.88 (14)	N2—C10—C11—C16	−109.81 (15)
C4—C5—C6—O2	−179.43 (14)	C16—C11—C12—C13	0.2 (2)
C4—C5—C6—C7	0.3 (2)	C10—C11—C12—C13	178.36 (14)
C8—N1—C7—C2	3.3 (2)	C11—C12—C13—C14	0.1 (3)
C8—N1—C7—C6	−176.38 (12)	C12—C13—C14—C15	−0.1 (3)
C3—C2—C7—N1	−176.96 (12)	C13—C14—C15—C16	−0.1 (3)
C1—C2—C7—N1	2.7 (2)	C14—C15—C16—C11	0.4 (3)
C3—C2—C7—C6	2.7 (2)	C12—C11—C16—C15	−0.4 (2)
C1—C2—C7—C6	−177.66 (12)	C10—C11—C16—C15	−178.65 (16)

### Hydrogen-bond geometry (Å, º)

**Table:**

*D*—H···*A*	*D*—H	H···*A*	*D*···*A*	*D*—H···*A*
N1—H1···S1^i^	0.87 (1)	2.63 (1)	3.493 (1)	174 (2)

Symmetry code: (i) −*x*+2, −*y*+2, −*z*+1.
